# The Status of Occupational Stress and Its Influence on the Health of Medical Staff in Lanzhou, China

**DOI:** 10.3390/ijerph191710808

**Published:** 2022-08-30

**Authors:** Dongsheng Zhu, Jinyu Wang, Yurui Zhao, Lu Yang, Jinxia Gao, Xuhong Chang, Sheng Li, Yanni Zheng

**Affiliations:** 1 School of Public Health, Gansu University of Chinese Medicine, Lanzhou 730000, China; 2 School of Basic Medical Science, Lanzhou University, Lanzhou 730000, China; 3 Lanzhou Municipal Center for Disease Control, Lanzhou 730030, China; 4 Department of Toxicology, School of Public Health, Lanzhou University, Lanzhou 730000, China; 5 Department of Public Health, The First People’s Hospital of Lanzhou, Lanzhou 730050, China

**Keywords:** medical staff, occupational stress, physical health, mental health, social health

## Abstract

This study aimed to understand the status quo of occupational stress and its impact on the health of medical staff and provide a theoretical basis for relieving occupational stress and improving the health status of medical staff. The occupational stress and health status of medical staff in 14 hospitals in Lanzhou were studied using a general questionnaire, Effort–Reward Imbalance questionnaire, and Self-Rated Health Measurement Scale. A total of 2169 participants were included in the analysis, and 59.4% of the medical staff experienced occupational stress. The results of the occupational stress survey showed that the prevalence of occupational stress among medical staff aged 40–50, with a master’s degree or above, senior professional title, working for 10–20 years, and working more than 48 h per week was higher than in the other groups. The health survey results showed that, compared with other groups, the scores of physical, mental, and social health were lower in medical staff with working years of 10–20 years and working hours of more than 48 h per week. The results show that working years and working hours per week affect not only the level of occupational stress but also physiological, psychological, and social health.

## 1. Introduction

Occupational stress refers to the physical and psychological stress caused by the imbalance between the objective needs and the adaptive ability of individuals under certain occupational conditions [1]. With the development of society and continuous advancement in science and technology, the work requirements for professional people are constantly improving, and the detection rate of occupational stress also increases. According to the World Health Organization (WHO), occupational stress is a worldwide epidemic [2]. Occupational stress not only harms the physical and mental health of the occupational population but also causes economic losses to enterprise and society. International occupational health psychology research and occupational disease law have made the impact of occupational stress on occupational group planning a key issue [3]. In China, studies on the occupational stress and psychological symptoms of oilfield operators [4], manufacturing employees, and road and bus drivers have shown that the higher the degree of occupational stress [5,6], the more obvious the psychological symptoms and the worse the mental health status. In the United States, research shows that the annual cost of treating diseases caused by occupational stress is USD 50 billion–1 trillion [7,8,9]. The United Kingdom loses millions of working days each year due to occupational stress disorder [8]. According to estimates by the International Labor Organization, the annual economic loss caused by occupational stress is approximately USD 300 billion [10].

Occupational stress is a recognized hazard in education, agriculture, fisheries, and forestry, affecting not only teachers, police, social workers, prison officers, and those working in call centers but also medical staff [11,12]. The health of medical staff is related to the quality of medical services, and the development of social health undertakings is closely related to the health of medical staff. In addition to heavy physical work, medical staff also have to face the contradiction between professional obligations, personal safety, and other psychological problems. In addition, medical staff have high work pressure, frequent interpersonal contact, irregular work and rest, and high occupational risk, thus becoming a high-risk group exposed to occupational stress. A survey of 3236 general practitioners in China showed that 313 (9.67%) had low occupational stress, 1028 (31.77%) had medium occupational stress, and 1895 (58.56%) had high occupational stress [13]. A survey of 256 dentists in Shaanxi Province of China showed that 34.4% of dentists had occupational stress [14]. In Vietnam, the results of a cross-sectional study on occupational stress in dermatology medical staff showed that 6.4% of medical staff had occupational stress [15]. A systematic review and meta-analysis of occupational stress and associated factors in health care professionals in Ethiopia showed that the pooled prevalence of occupational stress was 52.5 (95%CI: (47.03, 57.96)) [16]. Long-term exposure to these stressful environments has a certain impact on the physical, psychological, and social health of medical staff [17]. Physiological diseases include hypertension, neurasthenia, dyspepsia, and impaired immune function [18,19,20]. Psychological symptoms include depression, anxiety, and job burnout [21,22], which reduce employees’ coping ability. Social behavior abnormal is manifested as avoiding work, absenteeism, and medical accidents [23].

In addition, many factors cause occupational stress, mainly divided into occupational and personal factors. Occupational factors include working conditions, the working environment, and interpersonal relationships at work. Individual factors include gender, age, type A personality, self-perception, and ability to cope with pressure [2,24]. All these considerations indicate that medical staff with different demographic characteristics such as age and education level and occupation-related characteristics such as health-facility level, professional title, working years, and working hours per week perceive a certain degree of occupational stress, which has a certain impact on the physical and mental health of medical workers. To test this hypothesis, we assessed the occupational stress and health status of healthcare workers. Our assessment paid particular attention to their occupational stress and health status over different working years and working hours per week. We invited medical staff from 14 hospitals of different levels in Lanzhou to anonymously provide information on these topics through paper-based self-administered questionnaires.

At present, studies on occupational stress and health of medical staff in China are mainly concentrated in the economically developed areas in the central and eastern parts of China, and there are few studies in western regions such as Gansu province. Lanzhou is located in western China, and there are obvious differences in economic conditions and medical levels between Lanzhou and the central and eastern parts of China. Few studies have been conducted on the occupational stress and health of medical staff. This study discusses the occupational stress and health status of medical staff in Lanzhou by analyzing different demographic and occupation-related characteristics of medical staff and provides a theoretical reference for managers to take comprehensive measures to improve the work enthusiasm of medical staff and stabilize the medical staff.

## 2. Materials and Methods

### 2.1. Participants

**Figure 1:**
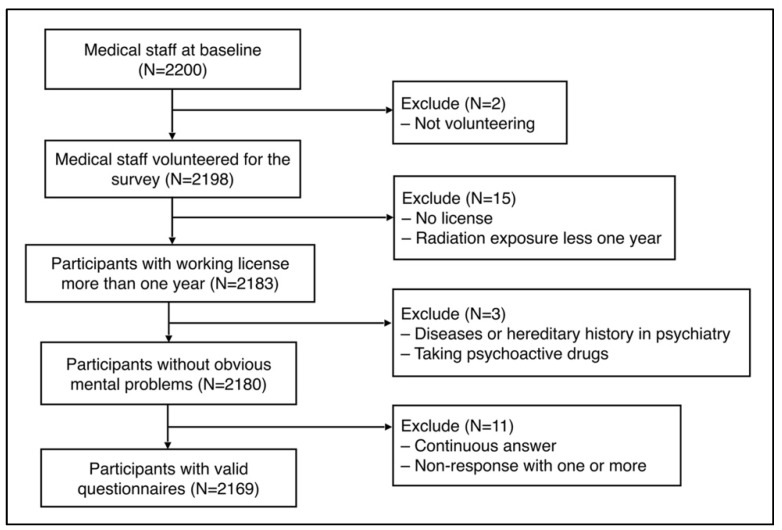
Definition of the study participants.

This is a cross-sectional study. A questionnaire survey was conducted among 2200 medical staff from 14 hospitals in Lanzhou from 1–31 December 2021. Stratified cluster sampling was carried out according to the different levels of health facilities in hospitals, which were divided into three groups: tertiary-level general hospitals, second-level general hospitals, and community health service center. An informed consent form explaining the questionnaire, survey purpose, and the principle of voluntary participation was distributed to the medical staff, and 2198 participants volunteered to complete the questionnaire survey. The study included 2183 participants with a physician or nurse qualification and more than 1 year of work experience. By on-the-spot inquiry during the distribution of questionnaires, workers with psychiatric diseases or a family history of such diseases and those taking psychoactive drugs were excluded. Based on the inclusion and exclusion criteria, 2180 medical staff were enrolled in this survey. A total of 2169 valid questionnaires were collected (99.5% response rate). Figure 1 presents a flowchart of participant selection.

### 2.2. Research Methods

A paper questionnaire was used to investigate the relationship between occupational stress and health.

#### 2.2.1. General Investigation

The general investigation included demographic and occupation-related characteristics. Demographic characteristics included sex, age, marital status, and educational level. Occupation-related characteristics included health-facility level, professional title, position, working years, and working hours per week.

#### 2.2.2. Occupational Stress Investigation

Based on Siegrist’s [25] Effort–Reward Imbalance (ERI) questionnaire model, the Chinese version of the ERI questionnaire compiled by Yang et al. [26] was selected. The questionnaire consisted of three sections, totaling 23 items, including effort (E, 6 items), reward (R, 11 items), and over-commitment (6 items). The Likert 5-level scoring method was adopted for each dimension, and the score of each dimension was added together. Taking the ERI index as the judgment standard, occupational stress was considered present when ERI was > 1; the higher the ratio of the ERI, the higher the level of occupational stress. An ERI > 1 indicates high effort–low reward return, indicating high occupational stress. An ERI ≤ l indicates a low effort–high reward return, indicating low occupational stress, and its calculation formula was ERI = E/(R × C), where C was the adjustment coefficient (the ratio of the number of items effort to the number of items reward). In this study, C = 6/11 [27]. Cronbach’s α coefficient for the overall internal consistency of this study was 0.795. The sampling fitness test (Kaiser–Meyer–Olkin, KMO test) was used for the validity analysis, and the KMO value was 0.873.

#### 2.2.3. Self-Rated Health Measurement

Self-rated health was measured using a questionnaire acquired from the Self-Rated Health Measurement Scale (SRHMS) developed by Xu et al. [28]. The scale consists of three subscales, 48 items, for the physical health scale, 17 entries; mental health scale, 15 entries; and social health scale, 12 items; each item, according to the different levels below the entry, was given a 10 cm line, and the respondents will be x on oneself think proper position, divided into 0–10 points. The subscale score was the sum of the positive and negative items of subordinates. The theoretical maximum scores of the physical health, mental health, and social health subscales were 170, 150, and 120, respectively. The higher the score, the better the health status [29]. The overall internal consistency of Cronbach’s α coefficient of this study was 0.947, and the subscales were 0.842, 0.888, and 0.933, respectively. The KMO value for validity analysis was 0.951.

### 2.3. Quality Control

The investigator was trained in advance to be familiar with the content of the questionnaire and matters needing attention to ensure the accuracy of the data. With the hospital department as the unit, the investigator uniformly distributed the questionnaire and explained in detail the purpose of the survey and the method of filling in the questionnaire so that the participants could fully understand and voluntarily participate in the survey to reduce the bias of no response and check and make up for the omission when the questionnaire was collected to ensure the integrity of the questionnaire. Two staff members were responsible for numbering, typing, summarizing, and proofreading the collected questionnaires. After typing, 5% of the questionnaires were selected, and the input quality was reviewed.

### 2.4. Statistical Methods

Data were double-entered into EpiData Entry version 3.1, and statistical analysis was performed using SPSS 26.0 (SPSS Inc., Chicago, IL, USA). The measurement data followed a normal distribution by the test of normality and were described by mean and standard deviation. The independent samples *t*-test was used for comparison between two groups, and one-way analysis of variance test was used for homogeneity of variance between multiple groups. For example, the self-rated health scores of medical staff with different demographic characteristics such as age and gender and occupational-related characteristics such as professional title and working years were analyzed. The counting data were expressed as the number of cases and composition ratio, and a chi-squared test was used for counting data, such as the occupational stress of medical staff with different demographic characteristics such as age and gender and occupational-related characteristics such as professional title and working years. Multiple linear regression analysis was used to analyze the factors influencing health status. Statistical significance was set at *p* < 0.05.

## 3. Results

### 3.1. Demographic Characteristics of Study Participants

A total of 2169 participants were included in the analysis; their characteristics are presented in Table 1. The results indicate that 73.8% (*n* = 1600) were female, 70.9% (*n* = 1538) were aged 30 years or older, 74.3% (*n* = 1162) were married, and 67.3% (*n* = 1460) had a bachelor’s degree.

### 3.2. Comparison of Occupational Stress Levels in Different Demographic Characteristics

The survey results showed that 59.4% (*n* = 1289) of the medical staff experienced occupational stress. The detection rate of occupational stress between the ages of 40 and 50 years was higher than that observed in the other age groups (*p* = 0.001). The detection rate of occupational stress with a master’s degree or higher was higher than that of the other groups (*p* = 0.002) (Table 1).

### 3.3. Comparison of Occupational Stress Levels in Different Occupational Characteristics

The detection rate of occupational stress with senior titles was higher than that of other groups (*p* < 0.001). The detection rate of occupational stress while working for 10–20 years was higher than that of the other groups (*p* = 0.013). The detection rate of occupational stress with working hours per week above 48 h was higher than that of the other groups (*p* < 0.001) (Table 2).

### 3.4. Comparison of SRHMS Score in Different Demographic and Occupational Characteristics

The higher the SRHMS score, the better the health status. Compared with the other groups, the scores of the physical health subscale were lower among medical staff aged more than 50 years, working in second-level general hospitals, working years of 10–20 years, and working hours of more than 48 h per week (all *p* < 0.05). Compared with the other groups, the mental health subscale scores were lower for medical staff aged 40–50 years, working in second-level general hospitals, working years of 10–20 years, and working hours of more than 48 h per week (all *p* < 0.05). Compared with the other groups, the social health subscale scores were lower in the 30–40 years age group, second-level general hospitals, 10–20 working years, and more than 48 h per week of working hours (all *p* < 0.05) (Table 3).

### 3.5. Comparison of the SRHMS Score among Medical Staff with an ERI Score >1 and ≤1

Medical staff with an ERI score >1 scored lower than those with an ERI score ≤1 on the physical, mental, and social health subscales, suggesting that occupational stress could affect the physical, mental, and social health of medical staff (all *p* < 0.001) (Table 4).

### 3.6. Exploration of Factors Influencing the Physical, Psychological, and Social Health of Medical Staff

Multiple linear regression was used to analyze the effects of different characteristics and occupational stress on physical, mental, and social health. Physical, mental, and social health scores were used as dependent variables, while age, health-facility level, professional title, working years, working hours per week, and ERI were used as independent variables. The assigned values are presented in Table 5. The results showed that age, health-facility level, working hours per week, and ERI affected the physical health of the medical staff (all *p* < 0.05). Older age, higher health-facility level, longer working hours per week, and higher ERI were factors associated with poorer physical health (Table 6). Health-facility level, working hours per week, and ERI affected the mental health of the medical staff (all *p* < 0.05). A higher health-facility level, longer working hours per week, and higher ERI were factors related to poorer mental health (Table 7). The working years, working hours per week, and ERI affected the social health of the medical staff (all *p* < 0.05). Longer working years, longer working weeks, and higher ERI scores were factors related to poorer social health (Table 8).

## 4. Discussion

Occupational stress is a special type of stress; although it is not like physical, chemical, and biological factors that can lead to a specific occupational disease, it can cause both physical nonspecific symptoms and psychological and behavioral changes, leading to impaired physical health [30]. With the increase in people’s demand for medical and health services, medical staff will inevitably experience occupational stress in the external environment, such as acute doctor-patient conflict and high work pressure in today’s complex medical environment, thus affecting their own health. We investigated the occupational stress levels of medical staff with different characteristics, and the survey results showed that 59.4% of medical staff experienced occupational stress, which was higher than the results of Zhang et al. [31] (53.08%) and Tsutsumi et al. [32] (57%). Studies have shown that long-term high occupational stress levels of medical staff lead to a series of work-related physical and mental health problems, such as musculoskeletal muscle diseases and anxiety and depression [33,34], which have a serious impact on the quality of life of medical staff [35,36].

ERI is one of the more mature tools for evaluating occupational stress. Its content covers job responsibilities, workload, salary, development prospects, social recognition, sustainable job composition, and initiative and has good reliability and validity in previous studies on occupational stress of medical staff [37,38]. In China, occupational health research focused only on civil servants, administrative personnel, and teachers in the past [39,40]. SRHMS is based on the WHO definition of health and, according to the social and cultural background in China, has high reliability and efficiency, making it more suitable for research regarding the health of all Chinese people [28].

The results of our study showed that the detection rate of occupational stress in the 40–50 years age group was higher than that of other age groups, and the mental health subscale score was lower than that of other groups, which was consistent with a survey report on occupational stress of nurses in China and the United States [41,42]. It may be that with the increase in age, the improvement of medical staff’s knowledge and experience, as well as the social family and patients’ demands and expectations on them, leads to higher occupational stress, which seriously affects their mental health.

Our research results show that the detection rate of occupational stress among medical staff with postgraduate education is higher than that of other groups, and the higher the education (degree) of medical staff, the greater the work pressure they experience, which is the same as the results of Xu et al. [43]. With the improvement of knowledge level, medical jobs have increasingly higher requirements on the knowledge and technical level of medical staff, and they can cope with the fierce knowledge competition, deal with the complex interpersonal relationship and the tense social environment by enhancing their competitiveness, which aggravates the occupational stress of highly educated medical staff [43]. Gao et al. [44] showed that doctoral students consider research pressure as their main work pressure, whereas medical personnel with other degrees consider work intensity as their main work pressure.

Our research results also showed that the detection rate of occupational stress among medical staff with senior professional titles was higher than that of the other groups. A study in China showed that the degree of occupational stress of clinical pharmacists with senior professional titles was significantly higher than that of other clinical pharmacists [45], and our research results were the same. This may be because, in China, most medical personnel with senior professional titles are middle-level managers, who have to not only treat patients at the clinical front line every day but also perform administrative work of the department well [46], which contributes to their high degree of occupational stress.

In addition, relevant studies show that with an increase in working years, the level of occupational stress increases, the degree of job burnout also increases [47], and the health status of medical staff is worse than that of medical staff with short working years [48]. However, our study showed that the medical staff with high occupational stress had a high proportion of 10–20 working years and had lower scores on the physical, psychological, and social health subscales compared with other groups, which was consistent with relevant research results [49,50]. The reason for this may be the length of the service period of the older medical staff, most as the backbone of our department staff, department in the hospital for the post rank are relatively high, bear the most basic medical department work tasks, in addition to treating patients in a day and doing a good job administrative department [41,46], which has a high degree of occupational stress, thus seriously affecting the medical staff’s physical, psychological, and social health.

The results of our study also showed that the medical staff working more than 48 h per week had a higher level of occupational stress and a lower score in the physical, mental, and social health subscales compared with other groups, which was consistent with the results in the US, UK, Germany, and Australia [51,52]. A longer working week for healthcare workers leads to fewer opportunities for group activities and less communication with family and friends. At the same time, working too much every week also affects the physiological cycle of medical staff, leading to a decrease in their working ability and efficiency, a decrease in their quality of life, and a failure to achieve the expected results, thus increasing the possibility of occupational stress and causing damage to their physical, psychological, and social health [53,54].

Finally, our study found that higher levels of occupational stress were associated with poorer health outcomes among healthcare workers, suggesting that reducing occupational stress among healthcare workers could improve their health outcomes. The results of multiple linear regression analysis showed that hospital level, weekly working hours, and occupational stress level affected the health status of the medical staff. The higher the health-facility level, the longer the working hours per week, the higher the level of occupational stress, and the worse the health status, consistent with relevant research results [55,56,57].

In summary, there were statistically significant differences in the composition of occupational stress among medical staff of different ages, education levels, professional titles, working years, and weekly working hours. In terms of self-rated health scores, there were significant differences in physical, mental, and social health scores among medical staff of different ages, health facilities, working years, weekly working hours, and ERI. A higher health-facility level, longer working hours per week, and higher ERI were factors related to poorer health.

This study conducted relevant investigations on medical staff in 14 hospitals in Lanzhou. However, there are still some limitations. First, as the Effort–Reward questionnaire and SRHMS are retrospective data, they have certain subjectivity and will produce certain information bias when filling in the questionnaire. Second, this study was a cross-sectional survey; therefore, the causal relationship between different demographic characteristics, occupation-related characteristics, occupational stress levels, and health status could not be obtained. Third, especially those medical staff with high levels of occupational stress were more likely to participate in studies on occupational stress, which may overestimate the occupational stress of medical staff.

Looking at the survey results, we were surprised that different working hours per week had a strong effect on occupational stress and health among medical staff. We decided to investigate the reasons for the longer working hours of health care workers and to repeat the survey regularly in this population, which would allow us to understand how occupational stress and health status change over time.

## 5. Conclusions

In conclusion, there were more medical staff members with high occupational stress in Lanzhou. Among them, working years and weekly working hours affect not only occupational stress levels but also physical, psychological, and social health. It is recommended that hospital and department managers arrange work reasonably according to the actual ability of medical staff, optimize the scheduling mechanism to appropriately reduce working hours, and actively give work support, encouragement, and appreciation. Psychological experts were regularly invited to give medical or psychological lectures to guide medical staff to maintain a positive psychological attitude so as to relieve their work pressure and better protect the physical and mental health of medical staff.

## Figures and Tables

**Table 1:** Comparison of occupational stress levels in different demographic characteristics. ERI: Effort–Reward Imbalance.

Variables	Total (*n* = 2169)	ERI > 1 (*n* = 1289)	ERI ≤ 1 (*n* = 880)	Chi-Squared Value	*p*-Value
Sex (%)					
Male	569 (26.2)	338 (59.4)	231 (40.6)	<0.001	0.998
Female	1600 (73.8)	951 (59.4)	649 (40.6)		
Age (%)					
<30 years	631 (29.1)	342 (54.2)	289 (45.8)	16.868	0.001
30–40 years	863 (39.8)	524 (60.7)	339 (39.3)		
40–50 years	455 (21.0)	300 (65.9)	155 (34.1)		
>50 years	220 (10.1)	123 (55.9)	97 (44.1)		
Marital status (%)					
Married	1612 (74.3)	979 (60.7)	633 (39.3)	4.702	0.095
Unmarried	508 (23.4)	281 (55.3)	227 (44.7)		
Divorced or widowed	49 (2.3)	29 (59.2)	20 (40.8)		
Education level (%)					
High school	65 (3.0)	41 (63.1)	24 (36.9)	15.310	0.002
Junior college	431 (19.9)	226 (52.4)	205 (47.6)		
Bachelor’s degree	1460 (67.3)	878 (60.1)	582 (39.9)		
Master’s degree or above	213 (9.8)	144 (67.6)	69 (32.4)		

**Table 2:** Comparison of occupational stress levels in different occupational characteristics. ERI: Effort–Reward Imbalance.

Variables	Total (*n* = 2169)	ERI > 1 (*n* = 1289)	ERI ≤ 1 (*n* = 880)	Chi-Squared Value	*p*-Value
Health-Facility Level (%)					
Tertiary-level general hospitals	1393 (64.2)	808 (58.0)	585 (42.0)	5.821	0.054
Second-level general hospitals	640 (29.5)	405 (63.3)	235 (36.7)		
Community health service center	136 (6.3)	76 (55.9)	60 (44.1)		
Professional title (%)					
Primary title	1063 (49.0)	584 (54.9)	479 (45.1)	21.240	<0.001
Intermediate title	685 (31.6)	429 (62.6)	256 (37.4)		
Senior title	347 (16.0)	234 (67.4)	113 (32.6)		
Others	74 (3.4)	42 (56.8)	32 (43.2)		
Working years (%)					
<10 years	1125 (51.9)	635 (56.4)	490 (43.6)	8.633	0.013
10–20 years	590 (27.2)	370 (62.7)	220 (37.3)		
>20 years	454 (20.9)	284 (62.6)	170 (37.4)		
Working hours per week (%)					
<40 h	1043 (48.1)	531 (50.9)	512 (49.1)	74.981	<0.001
40–48 h	658 (30.3)	412 (62.6)	246 (37.4)		
>48 h	468 (21.6)	346 (73.9)	122 (26.1)		

**Table 3:** Comparison of SRHMS score in different demographic and occupational characteristics.

Variables	Physical Health	Mental Health	Social Health
Age			
<30 years	132.44 ± 19.57	101.35 ± 21.74	86.53 ± 19.36
30–40 years	129.14 ± 20.83	98.06 ± 22.67	84.79 ± 19.63
40–50 years	128.15 ± 20.25	97.63 ± 23.33	86.38 ± 19.65
>50 years	127.09 ± 21.35	103.33 ± 24.77	89.18 ± 19.84
*f*-value	6.099	5.645	3.220
*p*-value	<0.001	<0.001	0.022
Health-facility level			
Tertiary-level general hospitals	131.00 ± 20.44	97.54 ± 22.78	86.52 ± 20.17
Second-level general hospitals	125.19 ± 22.45	95.98 ± 22.11	83.20 ± 20.42
Community health service center	131.62 ± 19.18	101.25 ± 22.99	87.35 ± 19.04
*f*-value	22.383	12.339	9.933
*p*-value	<0.001	<0.001	<0.001
Working years			
<10 years	131.16 ± 20.12	100.26 ± 21.80	85.42 ± 19.61
10–20 years	128.07 ± 21.35	96.94 ± 23.94	85.08 ± 19.94
>20 years	128.10 ± 19.95	100.76 ± 23.69	89.00 ± 18.92
*f*-value	6.153	5.020	6.460
*p*-value	0.002	0.007	0.002
Working hours per week			
<40 h	131.59 ± 20.01	102.08 ± 22.27	88.66 ± 18.79
40–48 h	128.32 ± 21.44	98.88 ± 22.77	83.76 ± 20.46
>48 h	127.35 ± 19.75	94.45 ± 23.33	83.57 ± 19.48
*f*-value	9.088	18.641	17.784
*p*-value	<0.001	<0.001	<0.001

**Table 4:** Comparison of the SRHMS score among medical staff with an ERI score > 1 and ≤1.

Occupational Stress Group	Physical Health	Mental Health	Social Health
ERI > 1	127.60 ± 20.25	95.16 ± 22.16	83.18 ± 19.31
ERI ≤ 1	132.73 ± 20.43	105.76 ± 22.37	90.32 ± 19.28
*t*-value	5.772	10.897	8.463
*p*-value	<0.001	<0.001	<0.001

**Table 5:** Assignment of factor-specific variables.

Variable	Name	Assignment
y1	Physical health	Accurate values
y2	Mental health	Accurate values
y3	Social health	Accurate values
x1	Age	0 = <30 years, 1 = 30–40 years, 2 = 40–50 years, 4 = >50 years
x2	Health-facility level	0 = tertiary-level general hospitals, 1 = second-level general hospitals, 2 = community health service center
x3	Professional title	0 = others, 1 = primary title, 2 = intermediate title, 3 = senior title
x4	Working years	0 = <10 years, 1 = 10–20 years, 2 = >20 years
x5	Working hours per week	0 = <40 h, 1 = 40–48 h, 2 = >48 h
x6	ERI	0 = ERI ≤ 1, 1 = ERI > 1

**Table 6:** Exploration of factors influencing the physical health of medical staff.

Variables	β	Standard Error	*t*-Value	*p*-Value	95%CI	
Intercept	129.994	2.238	58.075	<0.001	125.604	134.384
Age	−1.742	0.789	−2.209	0.027	−3.288	−0.195
Health-facility level	3.514	0.722	4.864	<0.001	2.097	4.930
Professional title	0.071	0.633	0.111	0.911	−1.171	1.312
Working years	0.130	0.946	0.137	0.891	−1.725	1.986
Working hours per week	−2.000	0.568	−3.521	<0.001	−3.114	−0.886
ERI	−4.294	0.899	−4.777	<0.001	−6.057	−2.531

**Table 7:** Exploration of factors influencing the mental health of medical staff.

Variables	β	Standard Error	*t*-Value	*p*-Value	95%CI	
Intercept	99.960	2.452	40.772	<0.001	95.153	104.768
Age	0.233	0.864	0.270	0.788	−1.461	1.927
Health-facility level	3.884	0.791	4.909	<0.001	2.333	5.436
Professional title	−0.054	0.694	−0.077	0.938	−1.414	1.306
Working years	0.120	1.036	0.115	0.908	−1.913	2.152
Working hours per week	−3.130	0.622	−5.031	<0.001	−4.350	−1.910
ERI	−9.575	0.985	−9.725	<0.001	−11.506	−7.644

**Table 8:** Exploration of factors influencing the social health of medical staff.

Variables	β	Standard Error	*t*-Value	*p*-Value	95%CI	
Intercept	85.392	2.126	40.165	<0.001	81.223	89.561
Age	−0.912	0.749	−1.218	0.223	−2.381	0.556
Health-facility level	2.419	0.686	3.525	<0.001	1.073	3.764
Professional title	−0.056	0.601	−0.093	0.926	−1.236	1.123
Working years	2.830	0.899	3.149	0.002	1.068	4.592
Working hours per week	−2.573	0.539	−4.769	<0.001	−3.630	−1.515
ERI	−6.470	0.854	−7.577	<0.001	−8.144	−4.795
